# Relation of Air to the House We Live in

**Published:** 1877-07

**Authors:** Max Von Pettenkofer


### Relation of Air to the House we live in.

At present the demands for ventilation in France sound very different from what they were about twenty years ago. They are now per hour and person :

| | | |
|---|---|---|
| Hospitals for ordinary cases - | 2,120—2,470 | cubic feet. |
| " wounded . . . | 3,53 ' | " |
| " epidemics . . | 5,300 | " |
| Prisons . . . . . . | 1,766 | " |
| Workshops, ordinary . . . | 2,120 | " |
| " unhealthy . . . | 3,530 | " |
| Barracks, day . . . . | 1,060 | " |
| " night . . . . | 1,410—1765 | " |
| Theatres . . . . . | " " | " |
| Large rooms for long meetings . | 2,120 | " |
| " " shorter " . . | 1,060 | " |
| Schools for children . . | 420— 530 | " |
| " adults. . . . . | 880—1,060 | " |

Such are the changes of times.

Now the many crevices, holes, and pores in our dwelling will no longer be considered by you as unlimited means of change of the air, since you know how large the change has to be ; you will rather feel anxious whence to procure such enormous quantities when you sit quietly within your four walls where you do not feel the least draught, where no curtain moves, and a feather lies quietly on the floor. This sensation of calm we owe to the insensibility of our nerves—*and yet the air moves.*

In order to give you some idea of the influences of differences of temperature of more or less well-shutting doors and windows, of a fire in a stove opening into the room, and of the partial opening of a window, I will give you shortly the results I obtained with the aid of the carbonic-acid measurement. The room had brick walls, and its size was 2,650 cubic feet.

With a difference of temperature of 34° Fahr (66° in- and 32° outside), the contents of the room changed once in one hour, equal to 2,650 cubic feet.

With the same difference, but a good fire in the stove, whose communication with the chimney was made as free as possible, the change of the air rose to 3,320 cubic feet, or about twenty-five per cent. When all openings, crevices in the windows and doors, were throughly pasted up, there was still a charge of 1,060 cubic feet per hour, or a fall of twenty-eight per cent. With a difference of temperature of 71° in- and 64° outside, the change amounted to 780 cubic feet only per hour. When opening a window of eight square feet, the change rose to 1,060 cubic feet per hour. these quantities are instructive. They show that a difference of temperature of 34° with carefully-cut openings and crevices is of greater influence than large communications with the outer air at a small difference of temperature.

The roaring fire and the draught of the stove produced only an increase of 700 cubic feet—one-third only of the necessary ventilation per head. I have examined a number of stoves opening in a room for a quantity of air which they abstract while the fire burns. The anemometer showed that it was never more than 3,105 cubic feet. Large wards in hospitals, schools, etc., heated by one open fireplace or stove, are sometimes wrongly believed to be well ventilated, because one perceives the air rushing into the same. But the main point is to know the quantity of required air and the quantity of outgoing air.

The free wall of a room of mine has been examined for its ventilating power. The room contained 2,650 cubic feet, and at 9.5° Fahr. difference of temperature between outside and inside, the spontaneous ventilation through each square yard amounted to about seven cubic feet, or forty-three gallons per hour.—*Dr. Max Von Pettenkofer, in The Popular Science Monthly for June.*

